# Laparoscopic Gastric Plication for the Treatment of Morbid Obesity: A Review

**DOI:** 10.1155/2012/696348

**Published:** 2012-07-03

**Authors:** Michael Kourkoulos, Emmanouil Giorgakis, Charalampos Kokkinos, Theodoros Mavromatis, John Griniatsos, Nikolaos Nikiteas, Christos Tsigris

**Affiliations:** ^1^Department of Surgery, Manchester Royal Infirmary, Manchester M13 9LL, UK; ^2^Department of Transplantation, Royal London Hospital, London E1 1BB, UK; ^3^Department of Surgery, Veroia General Hospital, Veroia 591 00, Greece; ^4^Department of Surgery, Evangelismos General Hospital, Athens 10675, Greece; ^5^Department of Minimally Invasive and Robotic Surgery, Athens University Medical School, Athens 11527, Greece

## Abstract

*Introduction*. Laparoscopic greater curvature plication is an operation that is gaining ground in the treatment of morbid obesity, as it appears to replicate the results of laparoscopic sleeve gastrectomy with fewer complications. *Aim*. Review of current literature, especially results on weight loss and complications. *Method*. 11 (eleven) published articles on laparoscopic gastric plication, of which 1 preclinical study, 8 prospective studies for a total of 521 patients and 2 case reports of unusual complications. *Results*. Reported Paracentage of EWL in all studies is comparable to Laparoscopic Sleeve Gastrectomy (around 50% in 6 months, 60–65% in 12 months, 60–65% in 24 months) and total complication rate is at 15,1% with minor complications in 10,7%, major complications in 4,4%. Reoperation rate was 3%, conversion rate was 0,2%, and mortality was zero. *Conclusion*. Current literature on gastric plication and its modifications is limited and sketchy at times. Low cost, short hospital stay, absence of prosthetic material, and reversibility make it an attractive option. Initial data show that LGCP is effective for short- and medium-term weight loss, complication and reoperation rates are low, and GERD symptoms are unaffected. More data is required, and randomized control trials must be completed in order to reach safe conclusions.

## 1. Introduction

Morbid obesity is one of the major health problems of the 21st century. Formally recognized by the WHO as a global epidemic in 1997, it was estimated that in 2008, 1.5 billion adults, 20 and older, were overweight. Of these, over 200 million men and nearly 300 million women were obese, with higher rates among women than men. The rate of obesity also increases with age at least up to 50 or 60 years old and severe obesity in the United States, Australia, and Canada is increasing faster than the overall rate of obesity. Once considered a problem only of high-income countries, obesity rates are rising worldwide and affecting both the developed and developing world. These increases have been felt most dramatically in urban settings [1].

Concurrently research on factors regulating obesity as well as possible treatments has been ongoing, with bariatric surgery making the greatest leaps and providing the means for better understanding of the metabolic and endocrine parameters involved in weight gain and weight loss [2, 3].

As the most effective means for excess weight loss available, bariatric surgery has been growing continuously, with more and more patients opting for surgical treatment of their condition, new operations and techniques being developed, and new instruments being produced. The advantages of minimally invasive surgery have been instrumental for this growth [4].

Many operations have been devised, with the Roux en Y gastric bypass being the most effective as far as excess weight loss is concerned, and sleeve gastrectomy being preferred by a growing number of surgeons due to its simplicity, effectiveness, and low rate of complications. In 2006, a new technique was presented, initially named total vertical gastric plication, better known today as laparoscopic greater curvature plication (Evidence Level III) [5]. Developed in Iran by Dr Talebpour as a cheap alternative to Laparoscopic Sleeve Gastrectomy, it appears to be gaining ground as its theoretical advantages of technical simplicity and low complication rate are of major importance to the growing industry that Bariatric Surgery has become, as well as to the industry of Bariatric Tourism.

## 2. Aim

Laparoscopic Greater Curvature Plication (LGCP) or Gastric Plication is a relatively new technique. Gastric Plication was initially proposed by Wilkinson and Peloso [6] in 1981 and introduced in 2006 by Dr Talebpour in Iran [5]. Operating in private hospitals throughout the country with scarce equipment, Dr Talebpour sought to develop a novel operation to mimic the well-established results of Laparoscopic Sleeve Gastrectomy, without the need to use costly equipment such as endoscopic staplers which were hard to come by. What he came up with was the LGCP which he initially named Total Vertical Gastric Plication, initially tested in animal models (especially sheep) and subsequently applying it to his volunteer patients. First results were published in 2006, and in 2007 a series of 100 consecutive patients were published, successfully placing LGCP on the map and adding it to the armament for the treatment of Morbid Obesity.

There is currently ongoing debate on the application of LGCP. The operation itself carries many potential advantages when compared to LSG, mainly due to the fact that there are no anastomotic lines and the risk of leak from a staple line is inherently inexistent. However, there are currently relatively few publications from authors performing the LGCP, resulting on very few data concerning the results as well as the complication rate of the LGCP, especially when compared to the LSG which has been extensively researched. This fact leads to distrust from the part of the international surgical community, and also led the American Society for Metabolic and Bariatric Surgery to issue a statement in March 2011, containing the following recommendations [7].

1. Gastric plication procedures should be considered investigational at present. This procedure should be performed under a study protocol with third-party oversight (local or regional ethics committee, institutional review board, data monitoring and safety board, or equivalent authority) to ensure continuous evaluation of patient safety and to review adverse events and outcomes.
2. Reporting of short- and long-term safety and efficacy outcomes in the medical literature is strongly encouraged. Data for these procedures should also be reported to a program's center of excellence database.
3. Any marketing or advertisement for this procedure should include a statement to the effect that this is an investigational procedure.

The aim of this study is to review current publications on LGCP especially reported complications and results on excess weight loss (EWL). No other published reviews on Gastric Plication or Laparoscopic Greater Curvature Plication were found during research of the literature, which makes this an original study.

## 3. Method

Online literature research and current library-based peer-reviewed journals review.

Online search engines employed are as follows: Medline/PubMed, Google Scholar, and SciVerse.

Key words are as follows: Gastric Plication, Laparoscopic Gastric Plication, Laparoscopic Greater Curvature Plication, (LGCP), and Total Vertical Gastric Plication, (TVGP).

Peer-reviewed journals: Surgical Endoscopy, Obesity Surgery, Surgery for Obesity and Related Diseases, Journal of Laparoendoscopic and Advanced Surgical Techniques, and Bariatric Times. Languages of the published data were: English and Spanish.

Timeframe searched was January 2000–December 2011.

## 4. Contemporary Literature Review

There are currently a total of 11 (eleven) articles in the published literature on LGCP as a standalone surgical technique. Publications referring to greater curvature plication in combination with another technique, such as Laparoscopic Gastric Banding with Greater Curvature Plication, were excluded. One variation of LGCP is Anterior Plication (AP), a technique proposed by Brethauer et al. involving plication of the anterior wall of the stomach without mobilization of the greater curvature. This study was included as there was also a group of patients undergoing LGCP, and also because omission of mobilization of the grater curvature could be an interesting idea, further reducing the risk of bleeding. A study by Khazzaka and Sarkis on a group of obese patients with gastroesophageal reflux disease (GERD) was also included as it shows the potential of gastric plication in treating these specific patients.

Research revealed 1 (one) preclinical study and 8 (eight) prospective studies.

## 5. Preclinical Studies

As was the case with LSG, LGCP was first performed to patients and subsequently studied in animal models. Data from Dr Talebpour's original animal trials have not been published.

 Menchaca et al. [8] performed a study on hound dogs, comparing the efficacy of 3 different methods for fastening the plicated gastric wall, namely, T-tags, sutures, and staples (Level of evidence II-1). The primary endpoint of the study was to compare the short-term durability of gastric plications and serosal bonds using a variety of fastening devices and techniques. Test subjects were euthanized at around 2 months after the operation, at which point necropsy was performed by a medical or veterinary pathologist. The durability of the infolded tissue was indicated by the persistence of the original fold geometry. The presence and degree of serosal bonding were noted. In the histopathologic evaluations, connective tissue bridging and angiogenesis were considered indicators of serosa-to-serosa healing. Erosive lesions and inflammatory tissue were noted when present. Tensile testing was performed using Instron tensile testing equipment. The sections of infolded tissue were securely gripped on either side of the fold and pulled apart at a constant rate until failure occurred. The force, displacement, and description of the failure were recorded and imaged. The authors found that all fastening devices and techniques created durable plication folds, except for the staple-suture combination. Intermittent point failures in serosal apposition occurred in those dogs that had received only 1 row of fasteners. In regions of the fold not containing fasteners, the serosal surfaces had not bonded. The durable plications were stronger than the surrounding tissue, with tissue failures often occurring in the tissues adjacent to the folds, but not within them. In all cases, the presence of a fold indicated the fold was strong enough to withstand the in vivo stresses created within the gastric wall from eating, normal gastric functions, and vomiting (if present). Pathology showed that the plication had healed, and new serosal tissue had bridged the opposing surfaces. Histologic studies of the bridges showed connective tissue networks and angiogenesis.

The authors concluded that the durability of the plication is dependent on continuous fixed serosal apposition by the fastening modality at multiple points along the fold, with multiple rows of fasteners, and fastener spacing of less than 2.5 cm within a row producing more durable outcomes.

## 6. Prospective Studies

### 6.1. Inclusion Criteria

An age over 18 years old and BMI > 40 or BMI > 35 accompanied by at least one comorbidity, according to the US National Institute of Health, criteria were applied in the studies of Skrekas et al. [9], Andraos et al. [10], Ramos et al. [11], Brethauer et al. [12], and Pujol-Gebelli et al. [13]. Inclusion criteria were not defined in the original Talebpour publication although minimum BMI was 36. They were also not defined in the Lopez-Corvala et al. study from Mexico [14], in which minimum BMI was 30. The inclusion criteria for the Khazzaka and Sarkis study included an age of 18–62 years and a BMI of 32–35 kg/m^2^ as well as a history of GERD and obesity for more than 5 years with unsuccessful attempts at conservative weight-loss therapy [15], as this study was aimed at demonstrating the efficacy of LGCP with Nissen fundoplication in obese patients with GERD.

### 6.2. Exclusion Criteria

Universal exclusion criteria varied with pregnancy, previous bariatric or gastric surgery, hiatal hernia, uncontrolled diabetes cardiovascular risks, a history of eating disorders, such as bulimia, medical therapy for weight loss within the previous 2 months, or any other condition that constitutes a significant risk of undergoing the procedure. A BMI > 50 was defined as an exclusion criterion for the Brethauer et al. and Skrekas et al. series.

### 6.3. Preoperative Preparation

In most studies, patients underwent upper GI endoscopy, blood tests, and abdominal ultrasound preoperatively. Anticoagulants were given 12 h preoperatively, and chemoprophylaxis with antibiotics was given with the induction of anesthesia [9]. Esophageal pH-metry was also performed in the Khazzaka and Sarkis study of the Obese-GERD group.

### 6.4. Surgical Technique

Patient positioning on the operating table is standard in all cases, in an anti-Trendelenburg position at 30-degree French position (operator between legs) and two assistants on each side of the patient. Trocar placement is also standard in all cases. Closed pneumoperitoneum is achieved using a five-trocar port technique similar to that employed in laparoscopic Nissen fundoplication. Trocar placement is as follows: one 10 mm trocar above and slightly to the right of the umbilicus for the 30° laparoscope; one 10 mm trocar in the upper left quadrant (ULQ) for passing the needle, for suturing, and for the surgeon's right hand; one 5 mm trocar also in the upper right quadrant (URQ) below the 10 mm trocar at the axillary line for the surgeon's assistant; one 5 mm trocar below the xiphoid process for liver retraction; and one 5 mm trocar in the upper left quadrant (ULQ) for the surgeon's left hand [10].

Ramos et al. preferred dissection of the angle of His as the first step of the operation, whereas in the larger studies of Skrekas et al. and Andraos et al. it was the final step of the dissection of the greater curvature of the stomach. Mobilization of the greater curvature is performed using either a LigaSure Vessel Ligation System (Covidien) or a Harmonic scalpel (Ethicon Endo-Surgery, Inc., Cincinnati, Ohio) initially by opening the greater omentum at the transition between the gastric antrum and gastric body. Once access to the posterior wall is achieved, the greater curvature vessels are dissected distally up to the pylorus and proximally up to the angle of His. Occasionally, posterior gastric adhesions are also dissected to allow optimal freedom for creating and sizing the invagination properly.

The next step is the introduction of a bougie which was of a diameter of 36 Fr in the Skrekas et al. study with 135 patients, and of 32 Fr in the studies of Andraos et al. and Ramos et al. with a total of 166 patients. Subsequently, the gastric plication is initiated by imbricating the greater curvature and applying a first row of extramucosal interrupted stitches which guided two subsequent rows created with extra-mucosal running suture lines. The first row stops 3 cm before the pylorus. The reduction results in a stomach shaped like a large sleeve gastrectomy. Choice of suture material varies amongst surgeons, (absorbable versus nonabsorbable) but all appear to be using multifilament sutures for the first row of interrupted sutures, and monofilament for the subsequent lines of running suture. Another important issue addressed by most authors, especially in the largest studies, is the distance between sutures, with all of them stressing the importance of a maximum distance of no more than 2 cm. Skrekas et al. modified their technique after 93 cases, and subsequently performed a double or even triple invagination creating a double mucosal fold on endoscopy. Reported results on this modification were similar in operating time and EWL with reduction of some complications resulting in shorter hospital stay [9]. An intraoperative methylene blue leak test was performed in most studies, with the exception of the Skrekas et al. study. No drains were placed in any of the cases.

In the Khazzaka-Sarkis group, Nissen fundoplication was performed after mobilization of the greater curvature, followed by plication of the body and antrum of the stomach.

### 6.5. Postoperative Management

In most studies, the patients underwent a gastrografin study on day 1 postoperatively, and immediately afterwards oral fluids were commenced. Skrekas et al. omitted the gastrografin study. Patients were discharged as soon as they were able to tolerate a liquid diet and were advised to progress to a soft diet after 15 days and to solid food after 30 days. Proton pump inhibitors and anticoagulation with low-molecular weight-heparin were prescribed regularly for 2 months and 14 days, respectively. During the first six postoperative months, all patients were treated with multivitamins and iron supplements. Follow-up visits were scheduled.

## 7. Results

Laparoscopic Sleeve Gastrectomy (LSG) has been in many ways the Holy Grail of Bariatric Surgery. A relatively simple technique, with short operating time, few complications, and very good results in Excess Weight Loss. LGCP is being proposed as a different way to reproduce the same results with even fewer complications. According to the Third International Summit on the status of LSG [16], these results are a reported mean percentage of excess weight loss at 1, 2, 3, 4, and 5 years of 62.7%, 64.7%, 64.0%, 57.3%, and 60.0%, respectively. The issue of coexistence of GERD or a hiatal hernia is a particular problem, as LSG has been recognized as a factor which worsens or even produces new onset of GERD symptoms (probably through a stasis mechanism). Based on a survey involving 88 surgeons who had performed 19605 LSG's, complications include staple-line leak, which is the most feared complication, at a rate from 0 to 10% (mean 1.3 ± 2.0) for high leaks at the level of the gastroesophageal junction, 0 to 10% (mean 0.5 ± 1.8) for lower leaks, 0 to 40% (mean 2.0 ± 5.0) for hemorrhage, splenic injury in 0 to 10% (mean 0.3, sd 1.3), liver injury in 0 to 7% (mean 0.2 ± 0.9), stricture in 0–5% (mean 0.6 ± 1.1), and other complications in 0 to 38% (mean 2.4 ± 8.4). Mortality rate was assessed at 0.1% with a standard deviation of 0.3.

In the 2011 Skrekas et al. Publication [9], 135 patients were studied (evidence Level III). Mean operative time was 58 min (45–80 min), mean hospital stay was 1.9 days (1–6 days), and mean followup was 22.59 months (8–31 months). Preoperatively, the group of patients had a mean Total Body Weight (TBW) of 113.3 ± 22.5 and a mean BMI of 39.5 ± 17.3. On followup, the percentage of excess weight loss (%EWL) was 51.7% at 6 months, 67.1% at 12 months, and 65.2% at 24 months. Postoperative mean TBW was 83.5 ± 17.3 and mean BMI was 29.6 ± 4.9. Inadequate weight loss (defined as less than 50% of the %EWL) was observed in 21.48%, with failure (%EWL of less than 30%) in 5.9% of the cases of inadequate weight loss. After subgroup analysis, the authors found that the results in weight loss were better in the group with a BMI of less than 45. Modification of their technique with formation of a double plication had no effect on weight loss. Total complication rate was 8.8% (12/135). Four patients presented nausea and vomiting which persisted for a few days. These patients were part of the single plication group. The authors comment that nausea, vomiting, and sialorrhea generally improved after modificating their technique to a double plication. Two patients presented with upper GI bleeding a few weeks after discharge. They were treated with endoscopic hemostasis. Two patients returning with general abdominal discomfort were found to have microleaks which were treated conservatively. Four patients had to be reoperated. One patient presented with portomesenteric thrombosis an the 24th postoperative day. The authors comment that portomesenteric thrombosis is a rare but serious complication of all laparoscopic operations, probably attributed to venous stasis due to pneumoperitoneum and anti-Trendelenburgs position [17]. The patient had jejunal necrosis and underwent jejunectomy. 1 patient was reoperated for gastric obstruction due to prolapse of the gastric fold, while two had accumulation of serous fluid within the cavity of the plication. These final cases led to the modification of their technique with creation of a double plication, thus creating smaller multiple gastric folds with less probability of both prolapse and accumulation of fluid. Mortality was zero. This is a very interesting study, the largest in literature so far, with relatively good medium term followup. The results on %EWL are similar to those achieved with LSG. Major complication rate is quite low (2.9%) and resulted in no mortality. The authors have presented a new modification to the standard technique of LGCP which could bear many benefits. Unfortunately they appear to be using the new technique in all new cases, instead of randomizing them in two groups of single-fold and multiple-fold technique. In any case, results presented in this study are very good with %EWL rates similar to those achieved with LSG for the 24 month follow-up period and low complication rates. Long-term follow-up results should be interesting.

Andraos et al. published a series of 120 cases [10]. Mean operative time was 65 minutes (45–90 minutes) and mean hospital stay was 36 hours (24 to 120). Most patients were discharged in 24 hours. There was one conversion due to intraoperative bleeding. Followup is very short, of only six months. Mean TWL in 1, 3 and 6 months is reported at 11.2 kg, 16 kg, and 23 kg, respectively, whereas %EWL at the same time is reported at 30.2% at 1 month, 43.9% at 3 months, and 48.58% at 6 months. No conclusions can be drawn on the effectiveness of LGCP from this study so far. Medium- and long-term follow-up results should prove useful. What makes this publication interesting is the very detailed description of complications. Intraoperative complications included one case of major bleeding due to mesenteric lesion from a faulty trocar, which resulted in laparotomy, suturing of the mesentery, and completion of the operation, and 9 cases of bleeding from the spleen, liver, or trocar insertion points, all of which were resolved by hemostasis without the need for conversion or transfusion. Early complications included 1 case of gastric obstruction due to fold invagination which had to be reoperated, and 1 case of gastric obstruction due to fold edema which did not respond to conservative treatment and also had to be reoperated, 5 cases of gastric obstruction due to fold edema which resolved with conservative treatment, 2 cases of food intolerance without obstruction which resolved with gastroscopy, 1 case of suture line rupture and herniation of the fold which resulted in a leak and had to be re-operated, 1 case of gastric fistula which was managed with laparoscopic suturing of the defect. There was 1 late complication, a patient presenting few months after the operation with upper GI bleed due to fold ulceration, treated with endoscopic hemostasis. What becomes evident is that gastric obstruction caused by the gastric fold is a recurrent theme. This makes the Skrekas modification of the LGCP even more interesting. Also, the authors of this publication are indirectly describing an algorithm for the management of gastric obstruction. Edema of the gastric wall always ensues after LGCP, and it could be the reason for most cases of postoperative vomiting. Therefore anti-inflammatory treatment should be given for a few days, along with PPI's, with gastrografin study performed before, after, or both. If the vomiting does not subside, one should attempt gastroscopy, since fold manipulation may improve the obstruction. In cases which do not improve, reoperation for refashioning and loosening of the plication should be the next step. This would relieve pressure within the stomach, reducing the probability of a tear caused by sutures and resulting in leaks, suture line rupture and herniation with possible necrosis and leak, and finally abdominal compartment syndrome, as presented by Watkins.

The classic study of Talebpour and Amoli from 2007 [5], which put LGCP on the map, included 100 patients. Mean preoperative BMI was 47 kg/m^2^ (36–58). Mean operative time was 98 minutes (70–152 minutes), and mean hospital stay was 1, 3 days (1–4 days). Mean followup was 18 months, and mean %EWL was 21.4% at 1 month, 54% at 6 months, 61% at 12 months, 60% at 24 months, and 57% at 36 months. Again, these results are similar to those achieved with LSG. Complications included 2 cases of hepatitis probably caused by medication in patients with fatty liver, 1 case of transient hypocalcemia due to inadequate intake, 1 case with persistent vomiting which on reoperation was attributed to a single adhesion causing a kink in the plicated stomach, 1 case of early postoperative leak attributed to high endogastric pressure due to persistent vomiting, 1 case of acute prepyloric gastric perforation far from the suture line, and 1 case of intrahepatic abscess 6 months after the operation caused probably by an intrahepatic hematoma and treated with laparoscopic drainage. Thirteen of the patients were diabetic and it appears that 6 months after the operation their diabetes was resolved. No further comments are made nor was there any further followup. Due to the low number of patients and scarce data, one should not venture in making a hypothesis of antidiabetic effects of the LGCP until further trials have been completed. Bearing in mind that the authors were sailing in uncharted waters at the time, and also the geopolitical status of Iran (ranitidine and antacids were given to the patients, probably due to lack of PPI's), one can only admire their efforts. What should be noted are the relatively long-term results (up to 36 months) showing an effectiveness comparable to that obtained with the LSG.

Lopez-Corvala et al. reported a series of 100 cases [14], operated in the Hospital Angeles in Tijuana Mexico. According to the authors, mean preoperative BMI was 39.7 (30–61), and %EWL was 43.1 at 1 month and 56.6 at 6 months. There were only 2 reported complications, one case of pulmonary embolism and one case of suture line disruption with perforation which led to reoperation and suturing. This study has many weaknesses, followup is very short, and complication rate appears to be extremely low, especially when compared to other studies of the same size. It appears that some patients with a BMI lower than 35 kg/m^2^ were included in the study. Although the senior author is a well-established bariatric surgeon, bariatric tourism could be involved and many patients could be lost to followup with their complications presenting and being treated in different countries. Again, %EWL is comparable to that achieved with LSG although the figures, especially on the 1 month followup, do seem a bit excessive.

Ramos et al., in their series of 42 cases [11], report a mean operative time of 50 minutes (40–100 minutes) and a mean hospital stay of 36 hours (24 to 96). Mean TWL on 1, 3, 6, 12, and 18 months from the operation was 10%, 15%, 22%, 28%, and 30%, respectively, with mean %EWL at 20% for the 1st month, 32% at 3 months, 48% at 6 months, 60% at 12 months, and 62% at 18 months. Only minor complications were observed, with symptoms such as nausea vomiting and sialorrhea up to 35% resolving spontaneously within 2 weeks. This small study shows very interesting results of %EWL, again comparable to LSG, but has the weakness of simplicity, small number of patients, and many patients lost to followup.

Khazzaka and Sarkis present a modification of LGCP specifically for patients with persistent GERD and a high BMI (30–35) [15], which is practically a Nissen fundoplication with plication of the rest of the stomach. They report a mean operative time of 65 to 95 minutes and a hospital stay of 24 hours for all patients. %EWL reached 58% at 12 months, while GERD symptoms, an esophagitis which were present in all patients, completely resolved. 7 of their patients presented transitory dysphagia, and none reported nausea. This is a small study with a small number of patients with a relatively low BMI. However, this may be a promising technique in this specific subgroup of patients. In fact most studies so far exclude patients with GERD or Hiatal Hernia (HH), Skrekas et al. state that they simply perform approximation of the crura, and the volume of the plicated stomach will keep it in place. Provided that LGCP will be proven an effective alternative, and given the fact that LSG is contraindicated in the presence of GERD or HH by most authors, randomized control trials will be required to prove whether simple approximation of the crura is effective without the need for a Nissen fundoplication. On the other hand, given the effectiveness of this technique in both treating GERD symptoms and esophagitis and weight loss, perhaps the international surgical community should consider offering it as a choice to patients undergoing surgery for GERD symptoms, who also have a BMI of 30–35 kg/m^2^.

The Pujol-Gebelli et al. is a small study with only 13 patients (Evidence Level III) [13]. Hospital stay was 5 days (3–21), and the authors report a %EWL comparable to that of LSG for the first 6 months. Of note is the fact that all patients presented nausea, vomiting, and sialorrhea postoperatively. 2 patients had to be reoperated, one for total dysphagia who was managed by refashioning of the plication, and the other for rupture of the suture line and herniation of the gastric wall through the sutures. In this case, a LSG was performed.

Brethauer et al. published their preliminary results from a pilot study [12]. With a total of 15 patients, the authors sought to obtain some insight on the question posed by Talebpour in his 2007 publication, whether an Anterior Plication would prove as effective as LGCP. Nine patients underwent AP with a mean operative time of 89 minutes (68–147), while 6 patients underwent LGCP with a mean operative time of 72 minutes (48–106). Mean hospital stay was 37 hours for both groups. %EWL after 12 months was 23.3% for the AP group and 53.4% for the LGCP group. The authors report 1 reoperation due to gastric obstruction. This is a very well-designed study despite the small number of patients included. Long-term followup is needed to determine the final impact of each operation on %EWL. One thing that becomes evident is the excellent %EWL in the LGCP group. On the other hand, initial results on weight loss in the AP group were discouraging. Two more important issues are raised by the authors. Firstly, there was no new onset or worsening of GERD symptoms. In fact, on follow-up gastroscopy, the gastric fold appears immediately below the LOS, and could function as a valve mechanism, reducing regurgitation of gastric contents into the esophagus. Secondly, the authors report unpublished data from an animal study, in which the reversibility of the LGCP is tested. In fact, the authors were able to reverse the LGCP and restore normal anatomy 2 months after the initial operation in all cases.

## 8. Discussion

Although the volume of published data so far is relatively small (a total of 521 patients included in the prospective studies), it would be safe to extract some conclusions.

LGCP appears to be an effective operation for the treatment of morbid obesity. All studies show a %EWL at the range of 50% on 6 months and 60% on 12 months. Studies with longer follow-up periods indicate a durable result for up to 36 months.

Complication rate appears to be low. In the 521 patients presented by the prospective studies, the rate of reported complications reaches 15.1% and reoperation rate was 3%. There was only 1 conversion (0.2%) due to a mesenteric injury from a faulty trocar, a rare but serious complication of laparoscopic surgery, and mortality was zero.

Minor complications were at a rate of 10.7%, with nausea, vomiting, and sialorrhea being the most common in 5.7%, intraoperative bleeding which was managed without the need for conversion or transfusions in 1,7%, and dysphagia or obstruction which was successfully managed conservatively in 2.6%.

Major complications presented at a rate of 4.4%. The ones managed conservatively included upper GI bleed managed with gastroscopy and endoscopic haemostasis in 0.6% and microleaks managed conservatively in 0.4%. Major complications that required reoperation were at a rate of 3%, the most common causes being gastric obstruction (due to fold prolapse, fold edema, adhesions, or accumulation of fluid within the gastric fold) in 1,5%, leaks due to suture line disruption and herniation in 0.7%, and gastric fistula in 0.1%.

No worsening of GERD symptoms or new GERD onset was reported; in fact there is reason to believe that LGCP could be the best operation in case of coexistence of GERD in an obese patient.

There are many lessons to be learnt from all the publications. The data currently available may not be representative as many patients could have an LGCP in foreign countries and not return for followup. Their weight loss and complications may never be studied. One recurrent theme in all studies is gastric wall edema, which may cause transient dysphagia, complete dysphagia, or even gastric compartment syndrome and perforation. One should be very careful when performing a tight plication as the ensuing edema could lead to serious complications [18]. In fact, most complications presenting with vomiting could be successfully treated with anti-inflammatories and PPI's in an attempt to reduce the edema. In more persistent cases, gastroscopy should be attempted as repositioning of the fold could relieve the obstruction. If that fails, reoperation is the only option. The Skrekas modification of the LGCP with formation of multiple smaller folds may prove a valuable alternative [9]. Suture line disruption with herniation and leaks are serious complications. Experimental data show that careful positioning of the sutures at a minimum distance of 2.5 cm, without penetration of the mucosa, produce a strong durable plication. Most materials have been proven effective for producing an effective plication and avoiding leaks [8].

The application of SILS and Robotic Surgery in LGCP are yet to be studied. Single Incision Laparoscopic Greater Curvature Plication could be viable, especially since there is no need for insertion of large caliber cumbersome staplers, or for extraction of a gastric specimen.

## 9. Conclusion

Current literature on Gastric Plication and its modifications is limited and sketchy at times. Low cost, short hospital stay, absence of prosthetic material, and reversibility make it an attractive option. Initial data show that LGCP is effective for short- and medium-term weight loss, complication and reoperation rates are low and GERD symptoms are unaffected. More data is required and randomized control trials must be completed in order to reach safe conclusions.

## Abbreviations:

LGCP: Laparoscopic greater curvature plication
AP: Anterior plication
TVGP: Total vertical gastric plication
LSG: Laparoscopic sleeve gastrectomy
BMI: Body mass index
TBW: Total body weight
%EWL: Percentage of excess weight loss
GERD: Gastroesophageal reflux disease
HH: Hiatal hernia
SILS: Single incision laparoscopic surgery.
